# Characterization of Membrane-Type Dissolution Profiles of Clinically Available Orally Inhaled Products Using a Weibull Fit and a Mechanistic Model

**DOI:** 10.1021/acs.molpharmaceut.2c00177

**Published:** 2022-08-08

**Authors:** Irès van der Zwaan, Frans Franek, Rebecca Fransson, Ulrika Tehler, Göran Frenning

**Affiliations:** † Department of Pharmaceutical Biosciences and the Swedish Drug Delivery Center (SweDeliver), Uppsala University, P.O. Box 580, 751 23 Uppsala, Sweden; ‡ Advanced Drug Delivery, Pharmaceutical Sciences, R&D, AstraZeneca, 43183 Gothenburg, Sweden

**Keywords:** dissolution, inhalation, mechanistic model, Weibull fit, Transwell

## Abstract

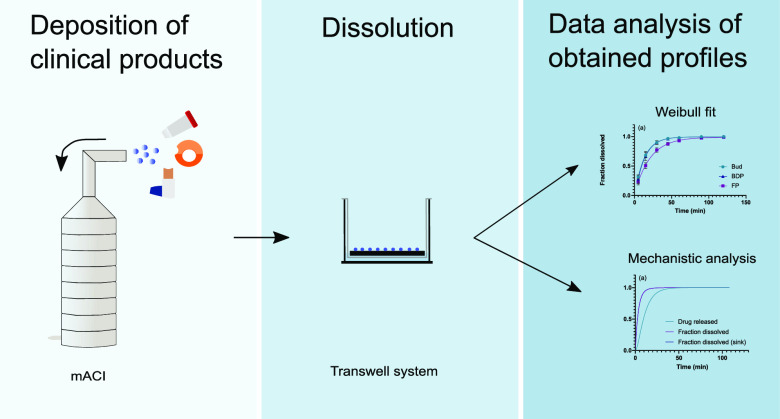

Dissolution rate impacts the absorption rate of poorly soluble inhaled drugs. In vitro dissolution tests that can capture the impact of changes in critical quality attributes of the drug product on in vivo dissolution are important for the development of products containing poorly soluble drugs, as well as modified release formulations. In this study, an extended mathematical model allowing for dissolution of polydisperse powders and subsequent diffusion of dissolved drug across a membrane is described. In vitro dissolution profiles of budesonide, fluticasone propionate, and beclomethasone dipropionate delivered from three commercial drug products were determined using a membrane-type Transwell dissolution test, which consists of a donor and an acceptor compartment separated by a membrane. Subsequently, the profiles were analyzed using the developed mechanistic model and a semi-empirical model based on the Weibull distribution. The two mathematical models provided the same rank order of the performance of the three drug products in terms of dissolution rates, but the rates were significantly different. The faster rate extracted from the mechanistic model is expected to reflect the true dissolution rate of the drug; the Weibull model provides an effective and slower rate that represents not only drug dissolution but also diffusion across the Transwell membrane. In conclusion, the developed extended model provides superior understanding of the dissolution mechanisms in membrane-type (Transwell) dissolution tests.

## 1 Introduction

When drug particles are inhaled, they will deposit in the lung and subsequently dissolve in the lung fluid before the drug substance can be absorbed across the lung epithelium and tissue to the systemic circulation.^1,2^ Dissolution is therefore a crucial process that can affect the absorption rate of a drug, and thus, it can be relevant for the in vivo performance.^3−5^ Because of this, in vitro dissolution tests that can capture the impact of changes in critical quality attributes of the drug product on in vivo dissolution are important for the development of products containing poorly soluble drugs, as well as modified release formulations.^1^ In vitro dissolution tests that are sufficiently discriminatory for critical quality attributes can be used to test batch-to-batch consistency of the same drug product and to evaluate the similarity of different products containing the same drug, that is, to evaluate bioequivalence. They can also be used to compare drug products containing different drugs.

Mimicking the lung in an in vitro test is a challenging task because of the uniqueness of some of the features of the lung. These features, such as the presence of lung surfactants and an extremely small volume of aqueous fluid, are difficult to reproduce, which makes the development of a standardized in vitro dissolution test challenging.^6−8^ Different approaches to determine the dissolution of orally inhaled drugs in vitro have been developed such as the Franz diffusion cell, the Transwell system, a flow-through apparatus, and a modified USP2 apparatus.^4,9−12^ One of these systems, the Transwell system, has been shown to successfully correlate in vitro dissolution data to in vivo absorption data.^12^ Dissolution takes place in a small volume of dissolution medium, thus mimicking the in vivo conditions. The small volume of dissolution medium also makes the Transwell method drug sparing, especially when used in combination with the modified Andersen Cascade Impactor (mACI) because drug deposition then occurs simultaneously on six filters.^12^ Hence, six independent dissolution experiments can be performed for each deposition (*n* = 6). The Transwell system consists of a donor compartment (where dissolution occurs) and an acceptor compartment (where samples are taken), which are separated by a membrane. Using such membrane-type dissolution tests entails the introduction of an additional diffusion rate parameter, as the drug needs to diffuse through the membrane from the donor to the acceptor compartment. This could have an influence on the obtained dissolution profiles that are measured using this type of tests. Additional factors, such as the limited volume of dissolution medium in the donor compartment and the amount and type of surfactants present, could also affect the in vitro dissolution profile.

The dissolution rate can impact the rate and extent of absorption of a poorly soluble compound or a modified release compound of the inhaled drug; therefore, it is of high importance that the dissolution profiles that are measured using a membrane-type dissolution test are well understood.^13^ Such an understanding can be achieved through mathematical modeling. More or less empirical expressions, such as the Weibull distribution function, are often used to analyze dissolution data.^12^ Although such analyses are highly useful, they provide limited insights into the underlying physicochemical processes. Additional information can be obtained from mechanistic models, in which all parameters are defined in terms of fundamental physical or chemical quantities.^14^ To this end, we have previously developed a mechanistic model that combines drug dissolution in the donor compartment with diffusional permeation through the membrane.^15^ For simplicity, a monodisperse powder was assumed. The modeling results exhibited an adequate correspondence with prior experiments and could be used to determine rate-limiting processes. However, as observed by May et al.,^16^ polydispersity can have a significant effect on the overall dissolution (and permeation) profiles, implying that this feature should be included in mechanistic modeling. In a recently published study by Amini et al., such a mechanistic model was indeed formulated in terms of binned particle-size data, where each bin corresponds to an impactor stage.^17^ This model utilizes and extends a methodology developed by Hintz and Johnson,^18^ who represented the underlying continuous particle-size distribution by a discrete distribution with 16 particles sizes and also considered permeation across a membrane, in a similar manner as in a Transwell dissolution setup. The Hintz and Johnson model is commonly used to describe dissolution in physiological based pharmacokinetic modeling and simulation.

The aim of this study was twofold. First, to extend our previously developed mechanistic model for membrane-type dissolution tests for inhaled drugs^15^ to polydisperse powders by using a novel methodology that retains the continuous particle-size distribution. Second, to demonstrate how such a mechanistic analysis can be used to minimize the effect of the membrane on dissolution data obtained from Transwell dissolution tests, thus providing improved estimates of important dissolution parameters. To this end, three commercial drug products are investigated. A semi-empirical model based on the Weibull distribution is used as reference.

## 2 Materials and Methods

### 2.1 Materials

Three drug products were purchased from Distansapoteket (Stockholm, Sweden) in their original clinical device, namely, Budesonide (Bud; Pulmicort Turbuhaler, 400 μg/dose, serial number: 20115200326688513272), Beclomethasone dipropionate (BDP; Beclomet Easyhaler, 200 μg/dose, serial number: 460790786871), and Fluticasone propionate (FP; Flutide Diskus, 500 μg/dose, serial number: 51M5NZPXEV). The corresponding pure APIs (Pharmaceutical Secondary Standard) were purchased from Sigma-Aldrich (Germany). Sodium dodecyl sulfate (SDS; at least 99% pure) and trifluoroacetic acid (TFA; at least 99% pure) were also obtained from Sigma-Aldrich (Germany). Organic solvents (at least HPLC grade) were purchased from VWR (France). Phosphate-buffered saline (PBS) tablets were obtained from EC Diagnostics (Uppsala, Sweden). Ultrapure water (PURELAB flex) was used.

### 2.2 Preparation of Media

PBS buffers were prepared by dissolving one PBS tablet in 1000 mL of water to obtain a 0.01 M phosphate buffer pH 7.4 with 0.14 M NaCl and 0.003 M KCl. Addition of 5 g (2 g) of SDS to 1000 mL yielded PBS buffers with 0.5% (0.2%) SDS. After addition of SDS, the pH of the buffers was measured as 7.2. The buffers were filtered through a 0.2 μm filter (Filtropur S 0.2 Sarstedt, Germany) before usage.

### 2.3 Solubility Determination

Determination of the solubility of Bud, BDP, and FP in PBS with 0.2 and 0.5% SDS was done by using the shake flask method. An excess of drug was added to 4 mL of medium and put on a shaking table (Heidolp Unimax 1010) for 72 h at room temperature. After 24 and 72 h, a 1 mL sample was taken and centrifuged (centrifuge 5430, Eppendorf, Germany) for 15 min at 14,500 rpm. An 800 μL sample was taken from the supernatant and analyzed using ultraperformance liquid chromatography-ultraviolet (UPLC-UV) (see Section 2.8) to determine the solubility. All solubility measurements were done in triplicate.

### 2.4 Particle-Size Determination

Particle-size determination of the formulations was done using a laser diffraction instrument (Coulter LS230, Coulter Corp, Miami, USA). Suspensions of the different formulations were made in the following manner. For Bud, 10 mL of water was added to 10 mg of powder from the Turbuhaler with 5 drops of 2% Tween 20 solution. For FP, 5 mL of water was added to 10 mg of powder from the Flutide Diskus with 5 drops of 2% Tween 20 solution. For BDP, 5–6 mL of water was added to 20–30 mg of powder from the Easyhaler with 5 drops of 2% Tween 20 solution. All suspensions were placed in a sonication bath (Ultrasonic Cleaner Branson, B5210E-MT, Danbury, USA) for 10 min and measured directly thereafter to prevent agglomeration. The particle-size distribution was measured with a particle refractive index of 1.333, an imaginary index of 1.0, and a dispersant refractive index of 1.332. Fraunhofer theory was used to calculate the particle-size distribution, in which PIDS data were included. Both the mass median diameter *d*_50_ and the span, (*d*_90_ – *d*_10_)/*d*_50_, where *d*_90_ and *d*_10_ are the 90th and 10th percentiles, were determined for all three formulations.

### 2.5 Dose Collection (mACI)

A modified Andersen Cascade Impactor (mACI) was used to deposit the drug particles on Whatman glass microfiber filters (21 mm, binder free, grade GF/C). The mACI was similar to a standard ACI up until stage 1. After stage 1, the stages and collection plates were replaced with five hollow stages, as previously described by Franek et al.,^12^ except that commercial drug products were used rather than a screenhaler to deliver the powder formulations. With a flow rate of 60 L/min, the cut-off diameter is 4.4 μm. For all three drug products, a flow rate of 60 L/min and a suction time of 0.3 s were used, as optimized by Franek et al.^12^ Devices were secured to the induction port using custom holders matching each device mouthpiece. The number of actuations was varied to achieve similar filter-doses for each drug product. The first and last few doses of each product were avoided in order to obtain as consistent depositions as possible. After each actuation, a sedimentation time of 20 min was implemented before another dose, or before collection of the filters.

The number of actuations was determined for all three drug products individually based on the strength of the formulation and the amount that ended up on the filter stage. The target amount of the drug on one filter was 5–10 μg.

### 2.6 Scanning Electron Microscopy

Scanning electron microscopy (SEM) images were taken using a Leo/Zeiss 1550 microscope (Jena, Germany) to visualize how the different drugs were deposited and dispersed. Each of the three different formulations were dispersed on a metal SEM holder with adhesive carbon tape on top by using the mACI. The SEM sample holders were placed in the filter stage of the mACI to mimic the filters that were used for the dissolution experiments. The same number of actuations was used for the SEM images as for the dissolution experiments. The SEM holders were then coated with a thin layer of Au/Pd under argon using a sputter coater (Polaron, Quorum Technologies Ltd., Newhaven, United Kingdom). An Inlens detector with a magnification of 500×, an acceleration voltage of 2.0 kV, and a working distance between 1.6–2.4 mm was used for all drugs.

### 2.7 Dissolution Method

To measure the dissolution from the drug particles that were deposited on the filters, the filters were transferred to a Corning Transwell system (24 mm inserts, polycarbonate membranes with 8.0 μm pore size, Sigma-Aldrich, Germany). The filters that were taken from the mACI were placed in the Transwell inserts with the deposited drug particles facing upward. The wells of the Transwell systems were prefilled with 2.3 mL of medium. The inserts with the filters were placed in the prefilled wells, and 700 μL of medium was added on top of the filters in the inserts to start the experiment. The Transwell plate was placed on a shaking table (Heidolph Unimax 1010) with a shaking speed of 150 rpm at room temperature. The dissolution method was based on prior work by Franek et al., to which the reader is referred for further details.^12^ The optimal shaking speed was selected based on the physical stability of the Transwell systems on the shaking table, without spilling the dissolution medium, and on the accuracy of the obtained data. Samples of 200 μL were taken at 5, 15, 30, 45, 60, 90, 120, and 180 min from the wells, and the samples were replaced with 200 μL of fresh buffer. After 180 min, 3 mL of methanol was added and stirred for an additional 30 min. A final sample of 200 μL was taken after these 30 min to determine the total amount of drug present in the Transwell system. The obtained samples were analyzed using a UPLC-UV instrument to quantify the amount of drug that diffused across the membrane (see Section 2.8) and to define the dissolution profile. Sink conditions were obtained when at least 3 times the amount of drug could be dissolved in the total volume of dissolution media. As a result of slow permeation across the membrane of the Transwell system, nonsink conditions may nevertheless prevail in the donor compartment, and a more conservative definition of sink conditions would thus be based on the smaller volume of this compartment.

To determine the diffusion through the membrane of the Transwell system, solutions of the model drugs in PBS with 0.5% SDS were also analyzed using the Transwell system. For this, the same procedure was used as described before, but instead of using filters with drug particles from the mACI, a solution of the desired drug was pipetted on a clean filter in the Transwell inserts to start the experiment. Sampling time points for the solutions were at 2, 5, 10, 15, 30, 45, 60, and 120 min. The amount of solution that was pipetted on the filters was chosen to resemble 10 μg of drug, which corresponded to 200 μL of 50 μg/mL stock solution of the model drugs in PBS with 0.5% SDS or acetonitrile, depending on the solubility of the model drug. All measurements were done with 6 replicates (*n* = 6).

### 2.8 Quantitative Analysis

For quantitative analysis, the samples were analyzed by using UPLC-UV.

A Waters Acquity UPLC-UV I-Class system with a BEH C18 column (2.1 mm × 50 mm) with 1.7 μm particle size was used to analyze the samples. Mobile phase A consisted of 0.03% TFA in water, and mobile phase B consisted of 0.03% TFA in acetonitrile. The method that was used to quantify Bud was adapted from Franek et al.,^12^ with a starting mobile phase composition of 65:35 (A:B) to 20:80 in 1.33 min and back to 65:35 with a total run time of 1.8 min. The flow rate for Bud was 0.6 mL/min, and the wavelength was set to 254 nm. To quantify BDP, an isocratic method with a mobile phase ratio of 45:55 (A:B), a flow rate of 0.8 mL/min, run time of 1.20 min, and a wavelength of 241 nm was used. For FP, an isocratic method with a mobile phase ratio of 50:50 (A:B), a flow rate of 0.9 mL/min, run time of 1.10 min, and a wavelength of 237 nm was used. The temperature of the column was set to 40 °C, the temperature of the sample compartment was set to 18 °C, and the injection volume was 2 μL for all samples.

Quantification was done by using a standard curve with an external standard. Validation of the UPLC-UV methods was done by the determination of inter- and intraday variation of standard curve samples in the range of 0.05 to 10 μg/mL.

### 2.9 Semi-Empirical Analysis of Dissolution Profiles

Dissolution data were analyzed using Microsoft Excel and plotted using GraphPad Prism 9. The Weibull distribution function was fitted to the dissolution profiles (fraction *u* of drug that has dissolved and permeated through the membrane vs time *t*),^12^
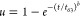
1where the scale and shape parameters are denoted by *t*_63_ and *b*, respectively

### 2.10 Mechanistic Analysis of Dissolution Profiles

In order to be able to analyze the dissolution profiles in mechanistic terms, our previously developed model^15^ was extended to include the effect of polydispersity. The resulting model thus accounts for dissolution, described by the Noyes–Whitney equation, and diffusional permeation through the membrane, as described by Fick’s law. Here and in the following, we use the term membrane to refer to the filter and the Transwell membrane.

#### 2.10.1 Dissolution of a Monodisperse Powder under Sink Conditions

Assuming sink conditions, the Noyes–Whitney equation can be expressed as
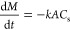
2where *M*(*t*) is the mass of drug remaining in the solid form at time *t*, *A*(*t*) is the total surface area of the solid drug, *C*_s_ is the solubility of the drug in the dissolution medium (mass per volume), and *k* is a dissolution rate constant, typically interpreted as the ratio between the diffusion coefficient of the drug and the thickness of a stagnant layer. For large particles or for small particles located close to a large structure, such as the membrane that separates the acceptor from the donor compartment in membrane-type dissolution tests, it is not unreasonable to consider *k* to be constant. It is nevertheless acknowledged that the Noyes–Whitney equation in general provides only an approximate (albeit often very useful) description of the dissolution process.^19^ Assuming monodisperse particles of size 2*r*(*t*) (initial size 2*R*), which retain their shape during dissolution, the surface area is proportional to the size squared and the mass is proportional to the size to the power of three, that is, *A* ∝ *r*^2^ and *M* ∝ *r*^3^. Eq 2 then implies that
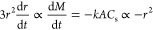
3that is, d*r*/d*t* = – *K*, where *K* is a constant. We may therefore write
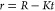
4so that the fraction *s*_sink_^mono^ of the drug that remains in the solid form can be expressed as
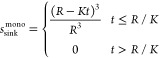
5

This is the Hixson–Crowell cube-root law^20^ (expressed in a slightly uncommon form).

#### 2.10.2 Dissolution of a Polydisperse Powder under Sink Conditions

We now consider dissolution of a polydisperse powder with a lognormal particle-size distribution *f*(*R*), that is,
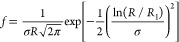
6where *R*_1_ is a scale parameter (the median) and σ is a shape parameter (the natural logarithm of the geometric standard deviation of the distribution). A lognormal particle-size distribution has also been used by Wang et al. to model the dissolution of polydisperse powders.^21^ According to eq 5, all particles smaller than *Kt* have dissolved at a certain time *t*. For the polydisperse powder, we may therefore express the fraction *s*_sink_^poly^ of the drug that remains in the solid form as
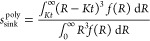
7

The result that emanates from this expression is provided in the Supporting Information (SI). In short, the fraction of the dissolved drug depends on two parameters, the shape parameter σ and a characteristic time for dissolution, denoted as . This characteristic time is defined so that the magnitude of the initial dissolution rate equals , where  is the initial value of *M*. This definition of  is consistent with the one used in our previous work.^15^ From eq 2, it can be seen that , that is,  equals the product of the dissolution rate constant *k*, the initial weight-specific surface area , and the solubility .

#### 2.10.3 Dissolution from a Polydisperse Powder under Non-sink Conditions

In order to extend the above results to the case when dissolution occurs in a donor compartment and drug subsequently diffuses across a membrane into an acceptor compartment, we use the same nondimensional variables as in our previous work.^15^ Hence, we introduce the nondimensional time *τ* = *t*/*t*_diss_, and the nondimensional “concentrations” of dissolved and solid drug, *c* = *C*/*S*_0_ and *s* = *S*/*S*_0_ together with the nondimensional solubility *c*_s_ = *C*_s_/*S*_0_. Here, *S*_0_ is the initial value of *S*, calculated as the ratio between the initial mass of drug and the volume of the donor compartment, implying that 1/*c*_s_ represents the ratio between the initial amount of the drug in the donor compartment and the maximal amount that can be dissolved without efflux, that is, a dose number for the drug in the donor compartment.^22^ It is assumed that all drugs are present in the solid form initially, so that *s*(0) = 1 and *c*(0) = 0. Conservation of mass can then be expressed as^15^
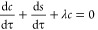
8

The fraction of the permeated drug *u* can be calculated as^15^
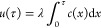
9where *x* is a dummy variable. The parameter λ is defined as the ratio between the characteristic time for dissolution, *t*_diss_, and a characteristic time for diffusion, *t*_diff_ (i.e., λ = *t*_diss_/*t*_diff_). The latter is defined so that the fraction of the permeated drug for purely diffusional permeation of dissolved drug becomes
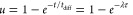
10

This is a consequence of eq 8 when *s* = 0. The fraction of permeated drug satisfies the equation^15^
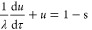
11

If we let *a* = *A*/*A*_0_ denote a nondimensional surface area of the drug (*A*_0_ is the initial value of *A*), the Noyes–Whitney equation takes the form
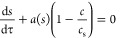
12

As indicated, the nondimensional surface area is a function of *s*. Equation 12 is a generalization of the one derived in our previous work^15^ for monodisperse powders to the polydisperse powders investigated here. As elaborated upon further in the SI, the separable structure of this equation implies that the amount of drug that dissolves under *non-sink* conditions in a small time interval dτ exactly matches the amount that dissolves under *sink* conditions in a small time interval dτ′, provided that these time intervals are related by dτ′ = (1 – *c*/*c*_s_)dτ. Moreover, as demonstrated in the SI, the fraction of the solid drug remaining after dissolution under *non-sink* conditions equals the fraction of the drug remaining after dissolution under *sink* conditions, provided the latter is evaluated at a retarded time τ′ defined as
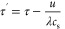
13

From eq 11, we thus obtain an equation for dissolution under nonsink conditions of the form
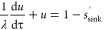
14where *s*_sink_^′^ denotes the *s*_sink_ evaluated at the retarded time τ′. As a check, we note that^15^
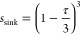
15for a monodisperse powder. Substituting τ′ for τ in eq 15, we thus obtain
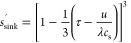
16

Hence eq 14 takes the form
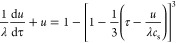
17in perfect agreement with the result obtained in our previous work.^15^ To obtain the fraction of the permeated drug for a polydisperse powder under non-sink conditions, we thus substitute *s*_sink_^poly^ evaluated at the retarded time τ′, as obtained from eq S10 in the SI and eq 13 for *s*_sink_^′^ in eq 14. To solve the resulting first-order nonlinear ordinary differential equation, we resort to numerical methods using the Cash-Karp (refined Runge–Kutta) method implemented in AlgLib.^23^

## 3 Results and Discussion

### 3.1 Collected Dose

A target amount of drug on one filter of about 5–10 μg resulted in 5 actuations for Bud using the Turbuhaler, 8 actuations for BDP from the Easyhaler, and 3 actuations for FP using the Diskus. The actual amounts that were deposited by the mACI on each filter are summarized in Table 1. To determine the diffusion rate across the membrane, a corresponding amount was added as a solution (Table 1).

**Table 1:** Arithmetic Mean (*n* = 6) of the Drug Added as Solution and Deposited as Powder for Budesonide (Bud), Beclomethasone Dipropionate (BDP), and Fluticasone Propionate (FP) in Diffusion and Dissolution Experiments^a^

	Bud (μg)	BDP (μg)	FP (μg)
Added as solution	9.8 (0.2)	9.2 (0.2)	10.8 (0.1)
Deposited as powder	11.6 (1.0)	6.7 (1.1)	7.4 (0.4)

a Standard deviations within parentheses.

### 3.2 Determination of Dimensional and Nondimensional Solubility

The solubility of all three compounds was determined in PBS with 0.5 and 0.2% SDS (Table 2). In PBS with 0.5% SDS, the solubility ranged from 878 μg/mL for Bud via 52 μg/mL for BDP to 13 μg/mL for FP. The model drugs thus provided a wide range of solubilities with Bud as the most soluble, then BDP, and the least soluble was FP. For future reference, the nondimensional solubility *c*_s_ is also reported in Table 2. The nondimensional solubility can be interpreted as the ratio between the amount of drug that can be dissolved in the donor compartment (without efflux) and the initial amount of the solid drug present. Hence, values of about 1 or smaller (as obtained for FP) indicate that solubility may be limiting, whereas values considerably exceeding 1 indicate sink conditions (as for BDP and in particular for Bud). One may equivalently consider 1/*c*_s_ as a dose number for the drug in the donor compartment, in which case, values of 1/*c*_s_ of 1 or larger indicate that solubility may be rate-limiting and values considerably lower than 1 indicate sink conditions. When the amount of SDS in the dissolution medium was reduced from 0.5 to 0.2%, the solubility of Bud and BDP reduced by slightly more than a factor of 2 and that of FP by almost a factor of 3 (Table 2). Sink conditions are commonly considered to prevail when at least 3 times the dose can be dissolved in the total volume of the dissolution medium. Hence, sink conditions prevailed in all cases except for FP in PBS with 0.2% SDS, in which only slightly more than two times the dose could be dissolved. The effect of this non-sink condition is expected to be small.

**Table 2:** Experimentally Determined Solubility of Bud, BDP, and FP^a^

Medium	Solubility	Bud	BDP	FP
PBS with 0.5% SDS	dimensional, *C*_s_ (μg/mL)	878 (5)	52 (1)	13 (0)
	nondimensional, *c*_s_ (−)	53	5.4	1.2
PBS with 0.2% SDS	dimensional, *C*_s_ (μg/mL)	399 (2)	23 (1)	4.7 (0)
	nondimensional, *c*_s_ (−)	36	2.4	0.5

a Standard deviations within parentheses (***n =*3**).

### 3.3 Particle-Size Distributions

**Figure 1:**
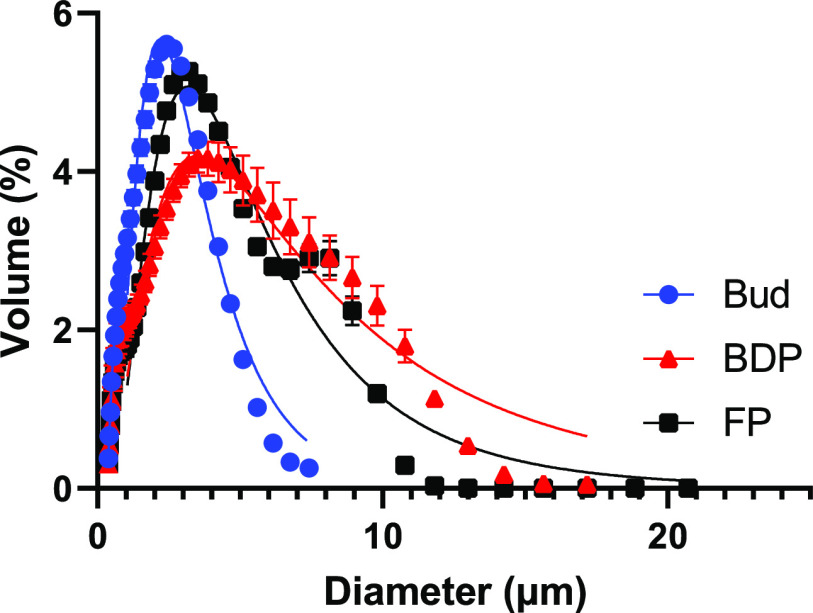
Particle-size distributions for Bud, BDP, and FP. Symbols represent experimental data and solid lines correspond to fits of the lognormal particle-size distribution (*R*^2^= 0.959, 0.896, and 0.9541 for BUD, BDP, and FP). Error bars indicate the standard deviation of two replicates for Bud and FP and three replicates of BDP.

Particle-size distributions for all three clinical formulations are shown in Figure 1. The mass median diameter ranged from 2.1 μm for Bud via 3.0 μm for FP to 3.3 μm for BDP. The span was comparable for all three formulations, ranging between 2.2 and 2.5 μm (Table 3). The solid lines in Figure 1 correspond to fits of lognormal particle-size distributions, from which the scale parameter *D*_1V_ (the median) and the shape parameter σ were extracted (Table 3). As seen in Figure 1, the lognormal distribution summarized the experimental data relatively well but generally underestimated the fraction of very small and overestimated the fraction of large particles. This is manifested in the values of *D*_1V_, which are considerably larger than the corresponding mass median diameters. For practical reasons, the presented particle-size distributions were determined for drug substance extracted from the products and not for drug deposited on the filters. Moreover, a standardized flow rate and suction time were used for all products. It is to be noted that only a fraction of the particles present in each drug product is expected to be deposited on the filters because there generally are considerable losses caused, for example, by insufficient deaggregation and impaction with parts of the device or the preseparator and incomplete sedimentation in the mACI. As a result, the particle-size distribution of the deposited drug may be somewhat truncated in comparison with the one presented in Figure 1, as the larger particles are lost in the upper part of the mACI and the sedimentation time is expected to influence the amount of smaller particles.^12^ The extracted parameters (in particular the shape parameter σ) are nevertheless considered to provide useful approximations.^24^ The lognormal distribution has the useful property that the same shape parameter σ characterizes both the particle-size distribution by volume (as in Figure 1) and by the number (as in our theoretical analysis presented above).^25^ The values of *σ* extracted from the fits can therefore be immediately used in subsequent dissolution and permeation modeling.

**Table 3:** Mass Median Diameter, Span, Scale Parameter *D*_1*V*_, and Shape Parameter σ of Bud (*n* = 2), BDP (*n* = 3), and FP (*n* = 2)^a^

Drug products	Mass median diameter (μm)	Span (μm)	*D*_1V_ (μm)	*σ* (−)
Bud	2.1 (0.0)	2.5 (0.6)	3.0	0.57
BDP	3.3 (0.2)	2.5 (0.1)	6.9	0.82
FP	3.0 (0.0)	2.2 (0.0)	4.9	0.67

a Standard deviations within parentheses.

### 3.4 Validation of Dispersion of Particles on Filter Stage

**Figure 2:**
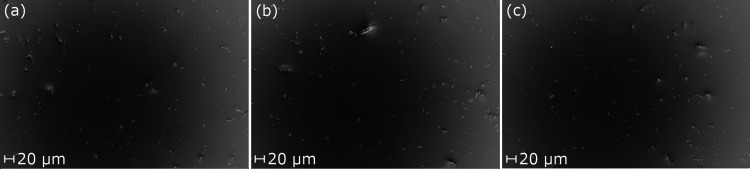
Scanning electron micrographs of deposition of (a) Bud, (b) BDP, and (c) FP.

To visualize how the particles were deposited on the filters, SEM images were taken from the filter stage of the mACI. The drugs look well dispersed regardless of the different inhalers and the different amount of doses that were used for different drug products (Figure 2a–c).

### 3.5 Determination of Diffusion Profiles and Diffusion Parameters

**Figure 3:**
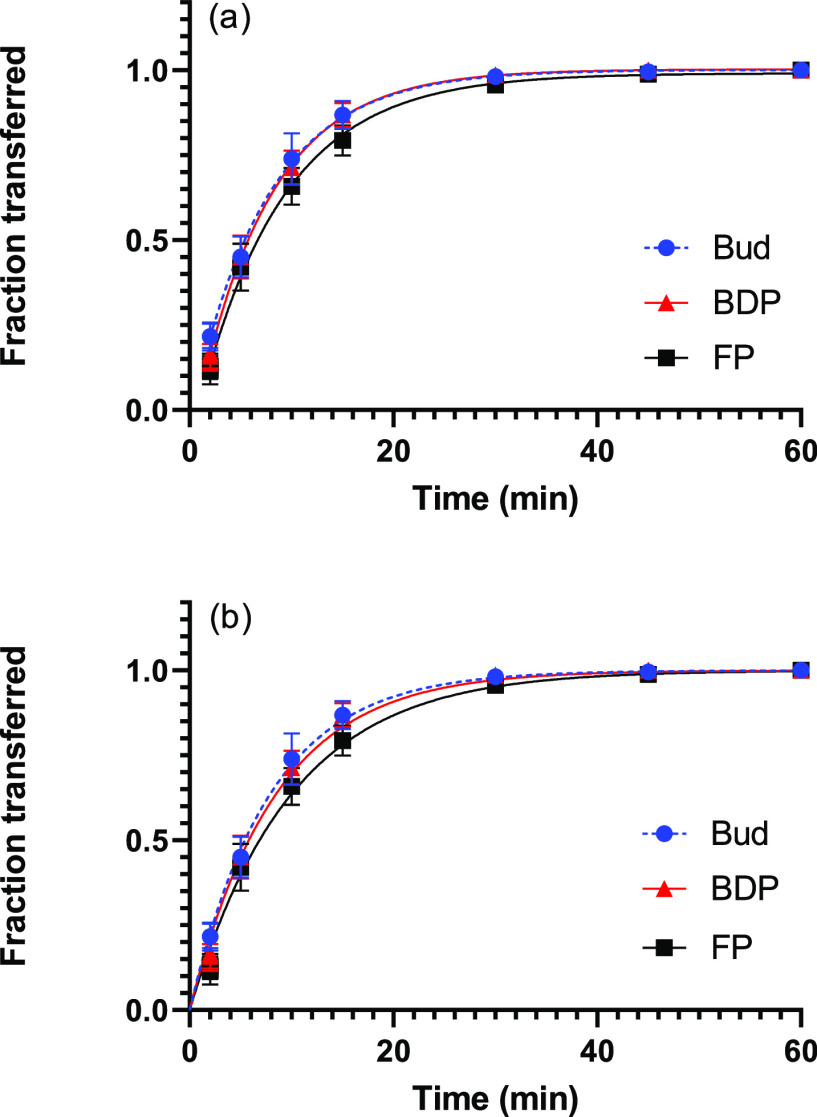
Diffusion profiles for Bud, BDP, and FP in PBS with 0.5% SDS. Solid lines in (a) represent fits of the semi-empirical model (*R*^2^ = 0.988, 0.996, and 0.980 for BUD, BDP, and FP) and in (b) of the mechanistic model (*R*^2^= 0.986, 0.982, and 0.980 for BUD, BDP, and FP). The error bars indicate the standard deviation of six replicates.

Before determining the dissolution profiles of the different drug products, the diffusion profiles of all drugs were determined in PBS with 0.5% SDS. No significant difference was observed between the diffusion profiles of the three model drugs, and all had a *t*_63_ of less than 10 min (Figure 3a, Table 4). Nevertheless, the *t*_diff_ (diffusion time) was determined for each drug individually using the diffusion profiles in PBS with 0.5% SDS. Bud had a diffusion time of 7.8 min, BDP of 8.4 min, and FP of 9.9 min (Table 4). A good fit was obtained between the mechanistic model and the experimental data for all three drugs (Figure 3b). The *t*_diff_ that was extracted using the mechanistic model shows high similarities with the *t*_63_ value that was extracted from the Weibull distribution. This is expected because the semi-empirical Weibull eq 1 reduces to the mechanistic eq 12 when *b* is close to unity. Both models yield a diffusion time under 10 min.

**Table 4:** Parameters Extracted from Diffusion Profiles for Bud, BDP, and FP in PBS with 0.5% SDS: Scale Parameter *t*_63_ Obtained by Fitting the Weibull Distribution Function, eq 1, and Characteristic Time for Diffusion, *t*_diff_ (*n* = 6)^a^

	Bud	BDP	FP
*t*_63_ (min)	7.7 (1.3)	7.9 (1.1)	9.3 (1.3)
*t*_diff_ (min)	7.8 (1.2)	8.4 (1.2)	9.9 (1.2)

a Standard deviations within parentheses.

### 3.6 Determination of Dissolution Profiles and Dissolution Time Parameters

**Figure 4:**
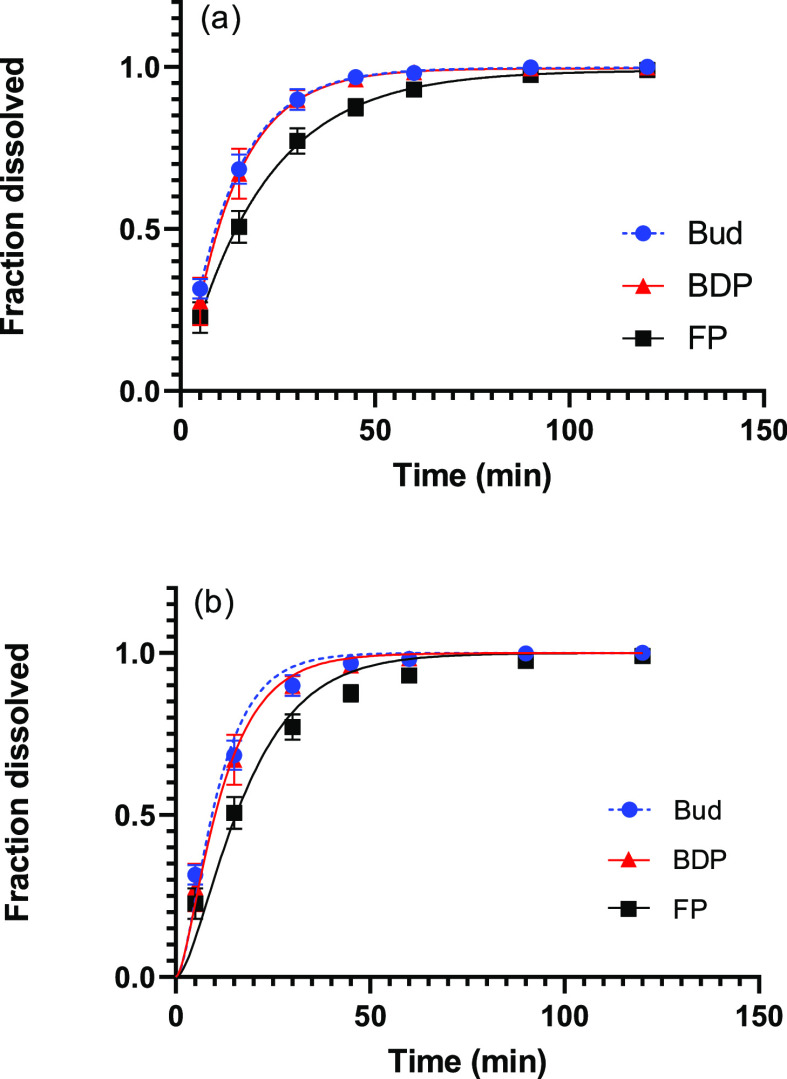
Dissolution profiles of Bud, BDP, and FP in PBS with 0.5% SDS. The solid lines in (a) represent fits of the semi-empirical model (*R*^2^= 0.991, 0.977, and 0.987) and in (b) of the mechanistic model (*R*^2^= 0.988, 0.996, and 0.980 for BUD, BDP, and FP). Error bars indicate the standard deviation of six replicates.

The dissolution profiles of all three drugs were determined in PBS with 0.5% SDS. Bud and BDP had a similar dissolution profile (Figure 4a) and their *t*_63_ values of around 13 min were not significantly different from each other (Table 5). FP dissolved significantly slower than Bud and BDP with a *t*_63_ of 20.8 min. As the solubility of the three drugs differed between all three, it was expected that the dissolution profiles would show a difference as well. However, there was no significant difference between the dissolution profiles of Bud and BDP. Prior absorption studies in man indicate that Bud and BDP have a similar mean absorption time of 0.6 h, which matches the obtained experimental dissolution data.^26,27^

**Table 5:** Parameters Extracted from Dissolution Profiles for Bud, BDP, and FP in PBS with 0.5% SDS: Scale Parameter (*t*_63_) and Characteristic Time for Dissolution (*t*_diss_)^a^

	Bud	BDP	FP
*t*_63_ (min)	13.0 (1.5)	13.5 (2.4)	20.8 (2.4)
*t*_diss_ (min)	3.6 (1.1)	2.9 (1.8)	6.1 (2.1)

a Standard deviations within parentheses (*n* = 6).

Further analysis of the dissolution profiles was done using the developed mechanistic model. In order to reduce the number of free parameters, the independently determined nondimensional solubility (*c*_s_; Table 2), shape parameter in the particle-size distribution (σ; Table 3), and characteristic diffusion time (*t*_diff_; Table 4) were adopted. This resulted in only one parameter that had to be determined by fitting the model to the experimental data, namely, the dissolution time (*t*_diss_). From the fits displayed in Figure 4b, *t*_diss_ was extracted as 3.6 min for Bud, as 2.9 min for BDP and as 6.1 min for FP (Table 5). The mechanistic model, with one free parameter, provides an adequate but not perfect description of the experimental data (Figure 4b). The observed deviations may have their roots in the less than perfect agreement between the theoretical and experimental particle-size distributions, which is especially pronounced for FP (Figure 1). The absence of any delay in the experimental data points toward a rapid initial dissolution, as would have been obtained from a significant fraction of very small particles, something that was not seen in the particle-size distributions. It is possible that the smallest particles dissolved in the 2% Tween 20 solution used for the particle-size analysis or that the particle-size distribution of the deposited particles differed slightly from those obtained for powder extracted from the drug products, thus skewing the results somewhat. This is to be expected because there are often considerable losses of particles caused, for example, by insufficient deaggregation, impaction with parts of the device or the preseparator, and possibly incomplete sedimentation in the mACI, implying that the population of particles on the filter will be different from the population of particles in each drug product.

The parameters extracted from the two models differ considerably. The semi-empirical Weibull distribution yields a *t*_63_ of around 13 min for Bud and BDP, and a significantly higher *t*_63_ for FP of roughly 20 min. However, the mechanistic model provides a *t*_diss_ of about 3 min for Bud and BDP and around 6 min for FP. Both the *t*_63_ values and the *t*_diss_ values exhibit the same order of the different drugs; both Bud and BDP have similar values, and FP is significantly higher.

The semi-empirical model (Weibull distribution analysis) uses no other input than the experimentally determined dissolution profiles and is therefore convenient to apply for routine studies to determine the rank order of drugs or drug products. However, the *t*_63_ values are effective parameters that not only depend on the rate of drug dissolution but also on the rate of diffusion of the drugs across the separating membrane. To assess drug dissolution per se, the dissolution profiles need to be corrected for the effects of drug diffusion across the membrane (see below). The mechanistic model, however, considers the solubility, particle-size distribution, and effects of drug diffusion across the membrane. In this way, the *t*_diss_ value that is extracted from the fits of the mechanistic model to the experimental data gives more insight into the actual dissolution time based on different input parameters. This adds additional value during drug product development and facilitates future translation of extracted in vitro dissolution data to the in vivo situation.

Similar benefits would result from an application of the model put forward by Amini et al.^17^ who extended the Hintz and Johnson^18^ model in two ways: First, nonsink conditions in the acceptor compartment were allowed and, second, sampling was explicitly accounted for. Our approach differs from the one used by Amini et al.^17^ in a number of ways. First, the underlying continuous particle-size distribution is retained. Hence, we are working in a framework not pioneered by Hintz and Johnson^18^ but rather related to the population-balance approach used by LeBlanc and Fogler.^28^ As a result, our account of polydispersity, presented in Section 2.10.2, and the transition from sink to nonsink conditions, elaborated upon in Section 2.10.3, are both novel. Second, we have assumed a time-independent stagnant-layer thickness, contrary to Amini et al.^17^ (as well as May et al.^16^ and Hintz and Johnson^18^) who considered a thickness that decreased with the particle size. The main motivation for using constant thickness is that the particles are lying on, and likely partly embedded in, the filter onto which they were deposited. It could therefore be argued that the relevant spatial length scale exceeds the particle size and that a constant stagnant-layer thickness therefore would be appropriate.^29^ Consistent with this, Amini et al.^17^ note that the stagnant layer, as a result of the mentioned factors, will be different from the one used by May et al.^16^ Third, we have considered the characteristic dissolution time to be a free parameter that absorbs effects resulting, for example, from the presence of micelles (see Section 3.7 below), incomplete wetting, hydrodynamics, and so forth. In the work by Amini et al.,^17^ a correction factor *F* was used to the same effect. The predictions by the two modeling approaches will nevertheless be similar (at least as long as sink conditions prevail in the acceptor compartment) because they are based on the same assumptions. In both cases, dissolution is described using the Noyes–Whitney/Nernst–Brunner equation^30−32^ and permeation of the drug across the membrane by Fick’s law.^33^ In our opinion, the modeling approach presented in this work therefore complements the one provided by Amini et al.^17^ The current state of knowledge does not allow one the luxury of rejecting either approach in favor of the other.

### 3.7 Mechanistic Analysis of the Dissolution Data

Using the characteristic diffusion time *t*_diff_ obtained from the diffusion profiles, correction of experimental data for the effects of drug diffusion across the membrane could in principle be done with the aid of the dimensional analogue of eq 11, which reads (notice that 1 – *s* is the fraction of the dissolved drug)
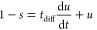
18

This step would either encompass numerical differentiation of the experimental data or differentiation of a semi-empirical function fitted to the data. For consistency, it is required that d*u*/d*t* = 0 initially because *s*(0) = 1 and *u*(0) = 0, implying that a more general function than the Weibull function used in this work would be needed. Alternatively, one can accept that a burst occurs that is not captured by the semi-empirical model.

Moreover, unless the nondimensional solubility *c*_s_ ≫ 1 (dose number 1/*c*_s_ ≪ 1), dissolution is generally hampered by limited solubility. This can be corrected for by using the dimensional analogue of eq 13 to calculate the retarded (or equivalent) time
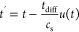
19such that a plot of 1 – s (which represents the fraction of the dissolved drug) vs *t*′ would correspond to equivalent dissolution under sink conditions. Although possible in principle, an analysis of the experimental data along these lines would depend strongly on the choice and behavior of the interpolation function near the origin and will therefore not be pursued further in this work. Rather, we discuss the results obtained from the developed mechanistic model.

**Figure 5:**
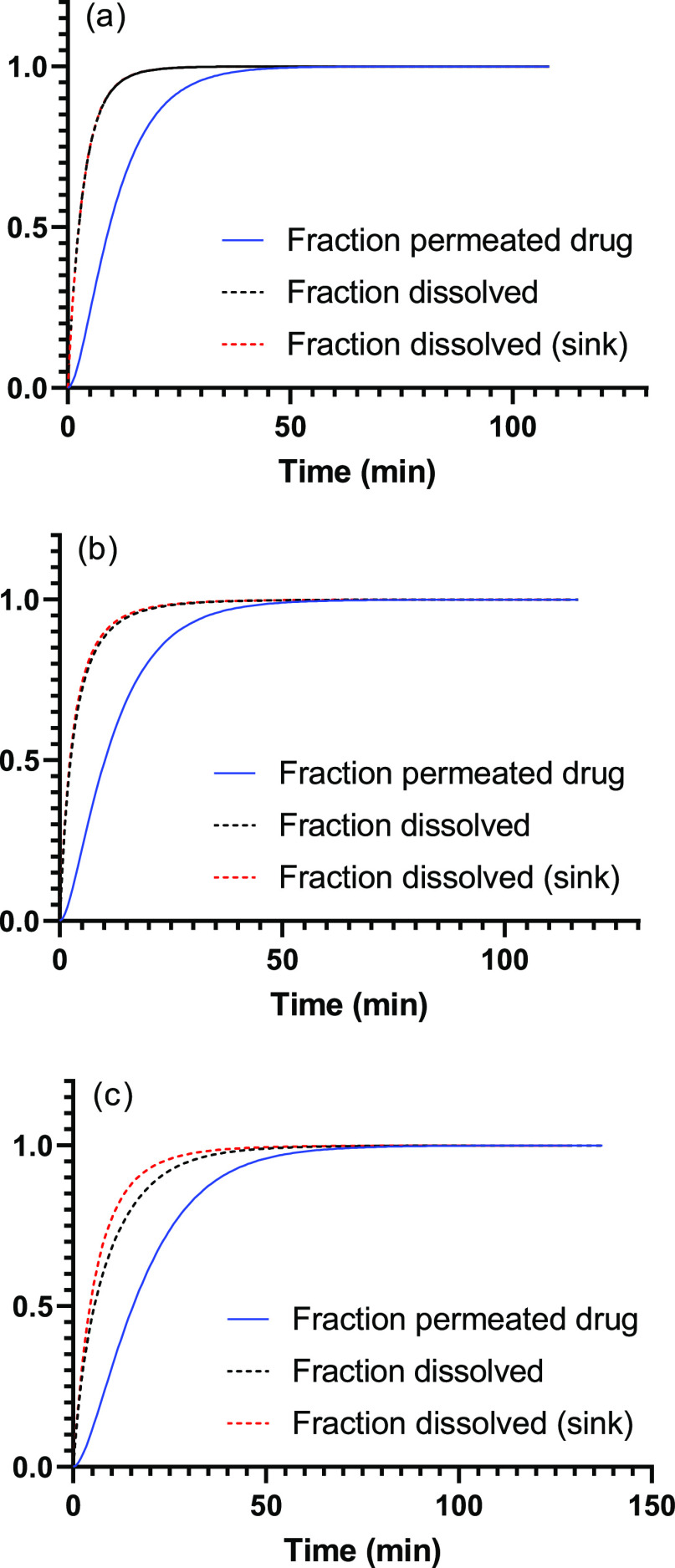
Fraction of permeated, fraction of the dissolved drug, and fraction of the dissolved drug that would have been obtained under sink conditions for (a) Bud, (b) BDP, and (c) FP.

Plotting the fraction of the permeated drug (*u*) and the fraction of the dissolved drug (1 – *s*) as a function of time, and in addition, the fraction of the dissolved drug (1 – *s*) as a function of the retarded time (*t*^’^, corresponding to dissolution under sink conditions) showed that there is a significant effect of the membrane on the fraction of the permeated drug (Figure 5). According to the mechanistic model, 63% of the Bud and BDP is dissolved in about 3.5 min, which is considerably shorter than about 13 min as obtained from the *t*_63_ of the Weibull model. Moreover, it can be concluded that sink conditions are apparent for both Bud and BDP, but are absent for FP. According to the mechanistic model, 63% of the FP is dissolved in about 8.5 min (6.5 min under sink conditions), which again is considerably more rapid than the 20 min obtained from the *t*_63_ of the Weibull model.

### 3.8 Prediction of Dissolution Data Using the Mechanistic Model

**Figure 6:**
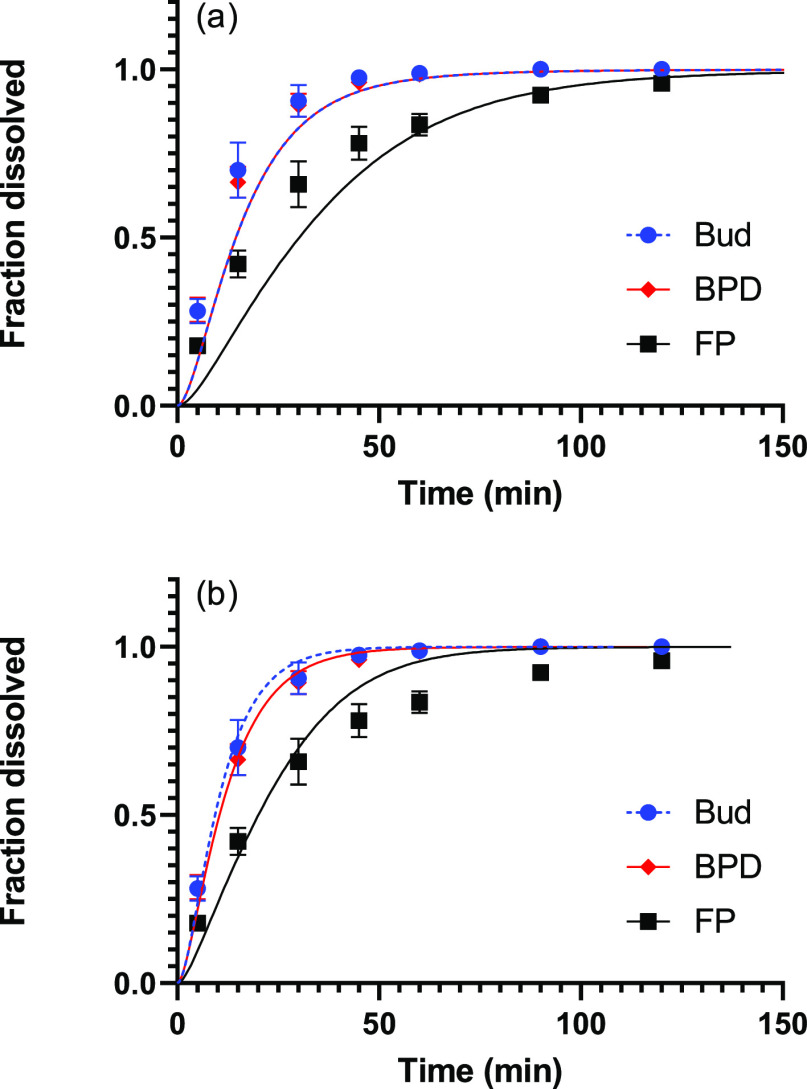
Comparison between observed (symbols) and predicted (curves) dissolution profiles for Bud, of BDP, and FP in PBS with 0.2% SDS calculated assuming (a) that the characteristic time for dissolution scales with solubility (*R*^2^ = 0.964, 0.952, and 0.925 for BUD, BDP, and FP) and (b) that the characteristic time for dissolution remains unchanged (*R*^2^ = 0.994, 0.994, and 0.956 for BUD, BDP, and FP). The error bars indicate the standard deviation of six replicates.

A decisive advantage of using a mechanistic model is that it can be used to predict the effect of changes in physicochemical parameters on the dissolution profiles. As an example, dissolution in a medium containing 0.2 rather than 0.5% SDS in PBS is considered, so that the solubility is lower for all drugs (Table 2). The effect on the dissolution profile is especially pronounced for FP because *c*_s_ is reduced to a value considerably smaller than 1 for this drug (symbols in Figure 6). Although a change from 0.5 to 0.2% SDS causes a reduction of the solubility by more than 50% for Bud and BDP, this reduction is not propagated to the dissolution profiles because *c*_s_ continues to be well above unity (symbols in Figure 6).

The solid lines in Figure 6a show model calculations based on the assumption that the characteristic time for dissolution scales with solubility according to the discussion following eq 9 (i.e., that *t*_diss_ is inversely proportional to *c*_s_), whereas σ and *t*_diff_ remain unchanged when reducing the amount of SDS in the dissolution medium. This would be the expected result for dissolution in a system without micelles, that is, without the kinetics resulting from solubilization. As seen, the correspondence between theory and experiments is not satisfactory. Similarly, the solid lines in Figure 6b show model calculations based on the assumption that all parameters except the solubility (i.e., σ, *t*_diff_ and *t*_diss_) remain unchanged. Such a situation would occur if surface kinetics is the rate-limiting step, which has been claimed to be common for dissolution of particles.^34^ More importantly, it has been claimed that it is the solubility in the continuous (water) phase rather than the total solubilizing effect of the medium that determines the dissolution rate in micellar systems, and this solubility is expected to stay constant.^35^ In this case, there is a satisfactory agreement between the experimental and theoretical permeation profiles, indicating that the assumptions underlying the mechanistic model are valid. However, some deviations are seen for FP at long times, as in PBS with 0.5% SDS, again likely because of deviations from the assumed particle-size distribution.

### 3.9 Rate-Controlling Mechanisms

A summary of the implication of our results is provided in Table 6. The membrane will inevitably have an effect on the measured dissolution profile unless diffusion across the membrane is considerably faster than dissolution (i.e., unless *t*_diff_ ≪ *t*_diss_ or 1/λ = *t*_diff_/*t*_diss_ ≪ 1). However, the dissolution test is nevertheless expected to provide meaningful results as long as diffusion does not dominate completely, that is, as long as 1/λ is not too large. In the current study, 1/λ ranged between 1.6 and 2.9 (Table 6), yet the same rank order between the dissolution rates were nevertheless obtained from the mechanistic and semi-empirical models. Analogous considerations apply for the dose number 1/*c*_s_ when dissolution occurs in a closed system. However, when the drug can diffuse out of the donor compartment, as for Transwell systems, sink conditions may be maintained also for high dose numbers provided that the diffusional permeation is rapid enough. The dose number 1/*c*_s_ nevertheless provides a convenient indication of whether sink conditions prevail or not. Sink conditions are obtained when 1/*c*_s_ ≪ 1 and effects of a limited solubility of the drug in the donor compartment may be seen otherwise. We emphasize that non-sink conditions do not invalidate membrane-type dissolution tests but adds a layer of complexity that should be understood. In agreement with the results presented in Figure 6, some minor effects of nonsink conditions are expected from BDP and more pronounced effects for FP in 0.5% SDS (Table 6). When the amount of SDS is reduced to 0.2%, the effects will be more pronounced for both compounds.

**Table 6:** Parameters That Determine the Relative Importance of Different Dissolution/Permeation Mechanisms: 1/λ = *t*_diff_/*t*_diss_ Indicates the Extent Of Retardation Caused by the Membrane, and 1/*c*_s_ Represents a Dose Number in the Donor Compartment

		0.5% SDS	0.2% SDS
	1/λ	1/*c*_s_	1/*c*_s_
Bud	2.17	0.02	0.03
BDP	2.90	0.19	0.42
FP	1.62	0.83	2.00

## 4 Conclusions

In this work, an extended model has been proposed for dissolution of polydisperse powders in a donor compartment and subsequent diffusion of the dissolved drug across a membrane into an acceptor compartment. Dissolution profiles of three drug products, determined using a Transwell dissolution test, have been analyzed using the proposed model and a semi-empirical drug dissolution model based on the Weibull distribution. Although the two mathematical models provided the same rank order of the studied drug products in terms of dissolution rates, the results enabled quantification of the effect of the membrane separating the donor and acceptor compartment on the experimental dissolution profiles. Moreover, effects of nonsink conditions were observed for the least soluble compounds. These findings add a layer of complexity to the analysis of experimental Transwell dissolution data but do not invalidate the method. A possible method to correct for these effects is outlined. From the mechanistic model, parameters indicating the extent of retardation caused by the membrane and the extent of sink conditions were defined. Finally, the model enabled the prediction of dissolution rates in different media.

## Supporting Information Available

Derivations related to dissolution of polydisperse powders and dissolution under nonsink conditions (PDF)
