# Supplementary material for: Characterization of Membrane-Type Dissolution Profiles of Clinically Available Orally Inhaled Products Using a Weibull Fit and a Mechanistic Model
Source: Mol Pharm. 2022 Aug 8;19(9):3114–24. doi: 10.1021/acs.molpharmaceut.2c00177 (PMC9449970; doi:10.1021/acs.molpharmaceut.2c00177)
Supplement: Supplementary file 1 — mp2c00177_si_001.pdf [file mp2c00177_si_001.pdf]

Supporting information for

Characterisation of membrane-type dissolution profiles of clinically available orally inhaled products using a Weibull fit and a mechanistic model

Irès van der Zwaan[1], Frans Franek[2], Rebecca Fransson[2], Ulrika Tehler[2], Göran Frenning[1,*]

[1]*Department of Pharmaceutical Biosciences and the Swedish Drug Delivery Center (SweDeliver), Uppsala University, P.O. Box 580, 751 23 Uppsala, Sweden*

[2]*Advanced Drug Delivery, Pharmaceutical Sciences, R&D, AstraZeneca, Gothenburg, Sweden*

*Corresponding author

Email: goran.frenning@farmbio.uu.se

Keywords: dissolution; inhalation; mechanistic model; Weibull fit; Transwell

S.1 Dissolution of polydisperse powders

In order to evaluate the expression that results from Eqs. (6) and (7) in the main text, we change variable to $x = \ln(R/R_1)$ with inverse $R = R_1 e^x$. Letting $\alpha = 1/(\sigma\sqrt{2})$, the lognormal particle-size distribution takes the form

$$f = \frac{\alpha}{R_1\sqrt{\pi}} e^{-(\alpha x)^2 - x} \tag{S1}$$

Noting that $\mathrm{d}R = R_1 e^x \mathrm{d}x$ and letting $g = -Kt/R_1$, the integrand in the numerator of Eq. (7), here denoted ϕ, can be expressed as

$$\phi = \frac{\alpha R_1^3}{\sqrt{\pi}}(e^x + g)^3 e^{-(\alpha x)^2} = \frac{\alpha R_1^3}{\sqrt{\pi}} \sum_{i=0}^{3} \binom{3}{i} g^{3-i}\, e^{-(\alpha x)^2 + ix} \tag{S2}$$

where binomial coefficients (with values 1, 3, 3 and 1 for $i = 0, 1, 2$ and 3) have been used to expand the product. To evaluate the integral, first use the identity

$$-\left(\alpha x - \frac{\beta}{2\alpha}\right)^2 + \left(\frac{\beta}{2\alpha}\right)^2 = -(\alpha x)^2 + \beta x \tag{S3}$$

and the definition[1] of the complementary error function erfc, to obtain

$$\int_h^\infty e^{-(\alpha x)^2 + \beta x} \mathrm{d}x = \frac{\sqrt{\pi}}{2\alpha} \exp\left[\left(\frac{\beta}{2\alpha}\right)^2\right] \operatorname{erfc}\left(\alpha h - \frac{\beta}{2\alpha}\right) \tag{S4}$$

where h is the lower limit of integration. Each term in Eq. (S2) is proportional to the integrand in Eq. (S4), with $\beta = i$. Hence the integral becomes

$$\Phi = \int_y^\infty \phi \,\mathrm{d}x = \frac{1}{2} R_1^3 \sum_{i=0}^{3} \binom{3}{i} g^{3-i} \exp\left[\left(\frac{i}{2\alpha}\right)^2\right] \operatorname{erfc}\left(\alpha h - \frac{i}{2\alpha}\right) \tag{S5}$$

where $h = \ln(Kt/R_1)$; c.f. Eq. (7) in the main text. Moreover, the denominator of Eq. (7) represents the 3rd moment of the lognormal distribution, which can be expressed as $R_1^3 e^{9\sigma^2/2} = R_1^3 e^{9/(4\alpha^2)}$, so that

$$s_{\mathrm{sink}}^{\mathrm{poly}} = \frac{1}{2} e^{-9/(4\alpha^2)} \sum_{i=0}^{3} \binom{3}{i} g^{3-i} \exp\left[\left(\frac{i}{2\alpha}\right)^2\right] \mathrm{erfc}\left(\alpha h - \frac{i}{2\alpha}\right). \tag{S6}$$

The time dependence comes from $g = -Kt/R_1$ and $h = \ln(Kt/R_1)$; hence $s_{\mathrm{sink}}^{\mathrm{poly}}$ can be expressed in non-dimensional form as a function of Kt/R_1. As in our previous work, we introduce a characteristic time for dissolution, denoted t_{diss}, from the requirement that the magnitude of the initial dissolution rate equals M_0/t_{diss}, where M_0 is the initial value of M. In other words, we demand that

$$\frac{1}{M_0}\left(\frac{\mathrm{d}M}{\mathrm{d}t}\right)_{t=0} = \left(\frac{\mathrm{d}s_{\mathrm{sink}}^{\mathrm{poly}}}{\mathrm{d}t}\right)_{t=0} = -\frac{1}{t_{\mathrm{diss}}}. \tag{S7}$$

Considering $g(t) = -Kt/R_1$ and $h(t) = \ln(Kt/R_1)$ as functions of time, we obtain the derivatives $g'(t) = -K/R_1$ and $h'(t) = 1/t$. The complementary error function is bounded, implying that the derivatives of the terms obtained for $i = 0$ and 1 vanish initially (for $t = 0$), since they contain the factor $g(0) = 0$. Moreover, for $i = 3$, we obtain the ratio between a Gaussian function and time. The argument of the Gaussian function contains $\ln t$, which tends to $-\infty$ in the limit $t \to 0$, implying that this term vanishes. For $i = 2$ we obtain a vanishing Gaussian function and a sole surviving term of the form

$$\left(\frac{\mathrm{d}s_{\mathrm{sink}}^{\mathrm{poly}}}{\mathrm{d}t}\right)_{t=0} = \frac{1}{2} e^{-9/(4\alpha^2)} \binom{3}{2} e^{1/\alpha^2} g'(0) \times 2\,. \tag{S8}$$

The factor 2 originates from the limit of the complementary error function obtained when its argument tends to $-\infty$. Upon using using $g'(0) = -K/R_1$ and substituting $\alpha = 1/(\sigma\sqrt{2})$ we finally obtain

$$\left(\frac{\mathrm{d}s_{\mathrm{sink}}^{\mathrm{poly}}}{\mathrm{d}t}\right)_{t=0} = -3\, e^{-5\sigma^2/2} K/R_1 = -\frac{1}{t_{\mathrm{diss}}} \tag{S9}$$

where the last equality follows from Eq. (S7). Hence $K = R_1 e^{5\sigma^2/2}/(3t_{\mathrm{diss}})$. Upon defining $\tau = t/t_{\mathrm{diss}}$, this yields $g = -e^{5\sigma^2/2}\tau/3$ and $h = \ln(\tau/3) + 5\sigma^2/2$. Since $\alpha = 1/(\sigma\sqrt{2})$, Eq. (S6) takes the form:

$$s_{\mathrm{sink}}^{\mathrm{poly}} = \frac{1}{2}\sum_{i=0}^{3}\binom{3}{i}\exp\left[\frac{(i^2 - 5i + 6)\sigma^2}{2}\right]\left(-\frac{\tau}{3}\right)^{3-i}$$
$$\times\ \mathrm{erfc}\left(\frac{2\ln(\tau/3)/\sigma + (5-2i)\sigma}{2\sqrt{2}}\right). \tag{S10}$$

This is the final result.

S.2 Dissolution under non-sink conditions

Note that Eq. (12) in the main text can be rephrased as

$$\frac{\mathrm{d}F}{\mathrm{d}\tau} = \frac{\mathrm{d}}{\mathrm{d}\tau}\int_1^s \frac{\mathrm{d}s}{a(s)} = \frac{1}{a}\frac{\mathrm{d}s}{\mathrm{d}\tau} = -\left(1 - \frac{c}{c_s}\right) \tag{S11}$$

where

$$F = \int_1^s \frac{\mathrm{d}s}{a(s)} \tag{S12}$$

is a monotonic and thus invertible function of s. Hence, dissolution from a given initial value of s during a small time interval $\mathrm{d}\tau$ under *non-sink* conditions corresponds to exactly the same change in F as does dissolution under *sink* conditions during a another time interval $\mathrm{d}\tau'$ given by

$$\mathrm{d}\tau' = \left(1 - \frac{c}{c_s}\right)\mathrm{d}\tau\,. \tag{S13}$$

Since $F(s)$ is invertible, the same is true for s and the assertion follows. Using Eq. (9) in the main text, Eq. (S13) can be integrated to give

$$\tau' = \tau - \frac{1}{c_s}\int_0^{\tau} c(x)\,\mathrm{d}x = \tau - \frac{u(\tau)}{\lambda c_s}. \tag{S14}$$

Hence, the fraction of solid drug remaining after dissolution under non-sink conditions equals the fraction of drug remaining after dissolution under sink conditions provided the latter is evaluated at the retarded time τ', i.e.

$$s_{\mathrm{nonsink}}(\tau) = s_{\mathrm{sink}}(\tau') = s_{\mathrm{sink}}\left(\tau - \frac{u}{\lambda c_s}\right). \tag{S15}$$

This important results is valid when (1) the dissolution rate is proportional to the difference between the drug solubility and the bulk concentration (i.e. to $C_s - C$) as in the Noyes-Whitney equation) and (2) the permeation rate is proportional to the bulk concentration (i.e., to C).

References

1 Abramowitz, M. & Stegun, I. A. *Handbook of mathematical functions*. (Dover Publications, Inc., 1965).
